# Spatial Variation of Leaf Chlorophyll in Northern Hemisphere Grasslands

**DOI:** 10.3389/fpls.2020.01244

**Published:** 2020-08-12

**Authors:** Yao Zhang, Ying Li, Ruomeng Wang, Li Xu, Mingxu Li, Zhaogang Liu, Zhenliang Wu, Jiahui Zhang, Guirui Yu, Nianpeng He

**Affiliations:** ^1^ Key Laboratory of Ecosystem Network Observation and Modeling, Institute of Geographic Sciences and Natural Resources Research, Chinese Academy of Sciences, Beijing, China; ^2^ School of Ecology and Nature Conservation, Beijing Forestry University, Beijing, China; ^3^ College of Resources and Environment, University of Chinese Academy of Sciences, Beijing, China; ^4^ Key Laboratory of Wetland Ecology and Environment, Northeast Institute of Geography and Agroecology, Chinese Academy of Sciences, Changchun, China; ^5^ Key Laboratory of Vegetation Ecology, Ministry of Education, Institute of Grassland Science, Northeast Normal University, Changchun, China

**Keywords:** allocation, chlorophyll, grassland, influencing factors, leaf nitrogen, spatial variation, trait distribution

## Abstract

Chlorophyll is the molecular basis for the function of photosystems and is also a promising tool for ecological prediction. However, the large-scale patterns of chlorophyll variation in grasslands remain poorly understood. We performed consistent measurements of chlorophyll *a*, *b*, *a*+*b*, and the *a*:*b* ratio (chlorophyll *a/b*) for 421 species across northern hemisphere grassland transects, recorded their distributions, variations, and influencing factors, and examined their relationships with leaf nitrogen. The results showed that the distributional ranges were 0.52–28.33 (mean 5.49) mg·g^−1^ dry weight, 0.15–12.11 (mean 1.83) mg·g^−1^ dry weight, 0.67–39.29 (mean 7.32) mg·g^−1^ dry weight, and 1.28–7.84 (mean 3.02) for chlorophyll *a*, *b*, *a*+*b*, and *a*/*b*, respectively. The chlorophyll averages differed among regions (higher in the Loess Plateau and the Mongolian Plateau than in the Tibetan Plateau), grassland types (desert grasslands > meadow > typical grasslands), life forms, life spans, and taxonomies. In the entire northern hemisphere grassland, chlorophyll concentrations and chlorophyll *a*/*b* were negatively correlated to photosynthetically active radiation and the soil N:P ratio, and positively correlated to the mean annual temperatures. These results implied that chlorophyll in grasslands was shaped by the layered structure of grasses, distinct plateau environments, and phylogeny. The allocation patterns of leaf nitrogen to chlorophyll differed among regions and grassland types, showing that caution is required if simply relating single leaf N or chlorophyll to productivity separately. These findings enhance our understanding of chlorophyll in natural grasslands on a large scale, as well as providing information for ecological predictive models.

## Introduction

Photosynthesis, the initiator of materials and energy cycles on Earth, can be divided into three continuous processes: 1) light harvesting; 2) zigzag electron transport, and 3) carbon fixation (the Calvin cycle). The first two processes require surrounding irradiation and occur *via* the plant photosystem (PS) on the thylakoid. There are two types of PS: PSI and PSII. Both are pigment-protein complexes; each includes a peripheral antenna (the light-harvesting Chl*a*/*b*-binding protein complex, LHC) that absorbs light and funnels its energy, and a reaction center (RC) for light-induced charge translocation. Chlorophyll (chl) is the most abundant pigment in the PS and has a key role in the conversion of sunlight into energy to initiate photo-biochemical reactions in plants.

The two most common types of chl in the PS of terrestrial plants are chl*a* and chl*b*. Both have strong light absorption and function mainly in the RC and LHC of photosystems in combination with proteins (Atsushi et al., 2018). Thus, concentrations of chl*a* and chl*b* are indicators of the light use efficiency and appear to be promising parameters describing the production capacity of plants (Croft et al., 2017; Li et al., 2018c). However, the amounts and combinations of the two types of chl are different in RC and LHC. For example, chl*b* occurs exclusively in LHC and is highly correlated with the peripheral antennae size, whereas chl*a* occurs mainly in the RC and the core parts of the antennae (Atsushi et al., 2018). That is, the relative amounts of chl*a* and chl*b* (i.e., the chl*a/b* ratio) are an indication of the allocation to the PS core and LHC, as well as of the plant’s functional balance between the efficiency of light capture and electron transport (Leong et al., 1985; Terashima and Hikosaka, 1995).

Plants may optimize productivity or sustain survival in different light intensities and qualities by adjusting chl concentrations and ratios. Generally, chl concentration increases in lower light, because increasing allocation to photochemical parts can enhance light harvesting and provide more energy for carbon fixation (Hallik et al., 2009). Chl*a*/*b* may be decreased in the shade compared with exposure to sunlight because of the larger peripheral antennae size (increased chl*b*) is conducive to enhancing the light harvesting under shade (Kitajima and Hogan, 2003). However, the evidence is inconclusive across scales, functional groups, and taxonomies (Hallik et al., 2009; Pollastrini et al., 2016; Li et al., 2018c). In addition, temperature and precipitation are important climatic factors related to plant growth, and their changes have multiple effects on plant functional traits, thereby affecting plant production capacity and adaptive strategies (Angela et al., 2014; Lv et al., 2016). Nonetheless, it has not previously been examined how chl is shaped by these environmental factors on a large scale.

Leaf N is thought to be a promising trait for productivity prediction because of its relationships with production-related traits (Bokhari, 1976; Wright et al., 2004) or gross primary production (Croft et al., 2015). However, several processes compete for leaf N, including the thylakoid reaction and carbon fixation, as well as between photosynthetic and non-photosynthetic activities. First, N is the most important component element in the PS core and antennae complexes, because it is necessary to synthesize chl molecules and combined protein complexes. Consequently, in theory, chl concentration should also be positively related to leaf N. The chl-leaf N relationship reflects the N cost of photosynthetic pigments. Second, leaf N plays crucial roles in the Calvin cycle in the form of photosynthetic enzymes, such as Rubisco, or other soluble proteins. The relationship between higher leaf N and higher photosynthesis and growth rates has been demonstrated in many studies (Wright et al., 2004; Wright et al., 2005; Shiflett et al., 2014). However, leaf N also plays key roles in persistence and resistance in plants living in harsh environments or under stress. It is argued that in deciduous forest, the relationship between leaf N and chl is weak owing to the seasonal N allocation dynamics between photosynthetic and non-photosynthetic fractions, which indicates the inaccuracy of using leaf N as a proxy for photosynthesis (Croft et al., 2017). In addition, the relationship between chl*a*/*b* and leaf N should be informative to describe N allocation to RC and LHC and the maintenance of their functional balance, but only a few studies have focused on a few species that have documented this relationship.

In recent years, large-scale ecological predictive technology has been based on chl fluorescence (Li et al., 2018a; Sun et al., 2018). However, it is still a huge challenge to provide informative chl parameters for ecological models because most existing studies focused on only a few species and sites. Little is known about the patterns of Chl in natural grassland communities at large scales, which undoubtedly hinders further technological developments (Sun et al., 2018). In the current study, we performed a field investigation of 30 sites across northern hemispheric grassland transects to reveal the distributions, variations, and influencing factors of chl. These sites included grasslands of three environmentally distinct plateaus (Mongolian, Loess, and Tibetan Plateaus—MP, LP, and TP, respectively) and three grassland types (meadow, typical grassland, and desert grassland—MD, TG, and DG, respectively). We analyzed chl*a*, chl*b*, chl*a*+*b*, and chl*a*/*b* for 421 species available in total and determined the relationships between leaf N and chl. The results are presented from the perspectives of the three plateaus and three grassland types to achieve a comprehensive understanding. We hypothesize that leaf N allocation to chl may differ among the three plateaus and grassland types due to distinct environments and resource limitations, and leaf N may not be closely related to chl in stressful grasslands such as Tibetan grasslands or desert grasslands because producing extra protective and resistant proteins under these stressful conditions will also compete for leaf N.HH

## Materials and Methods

### Study Area

**Figure 1:**
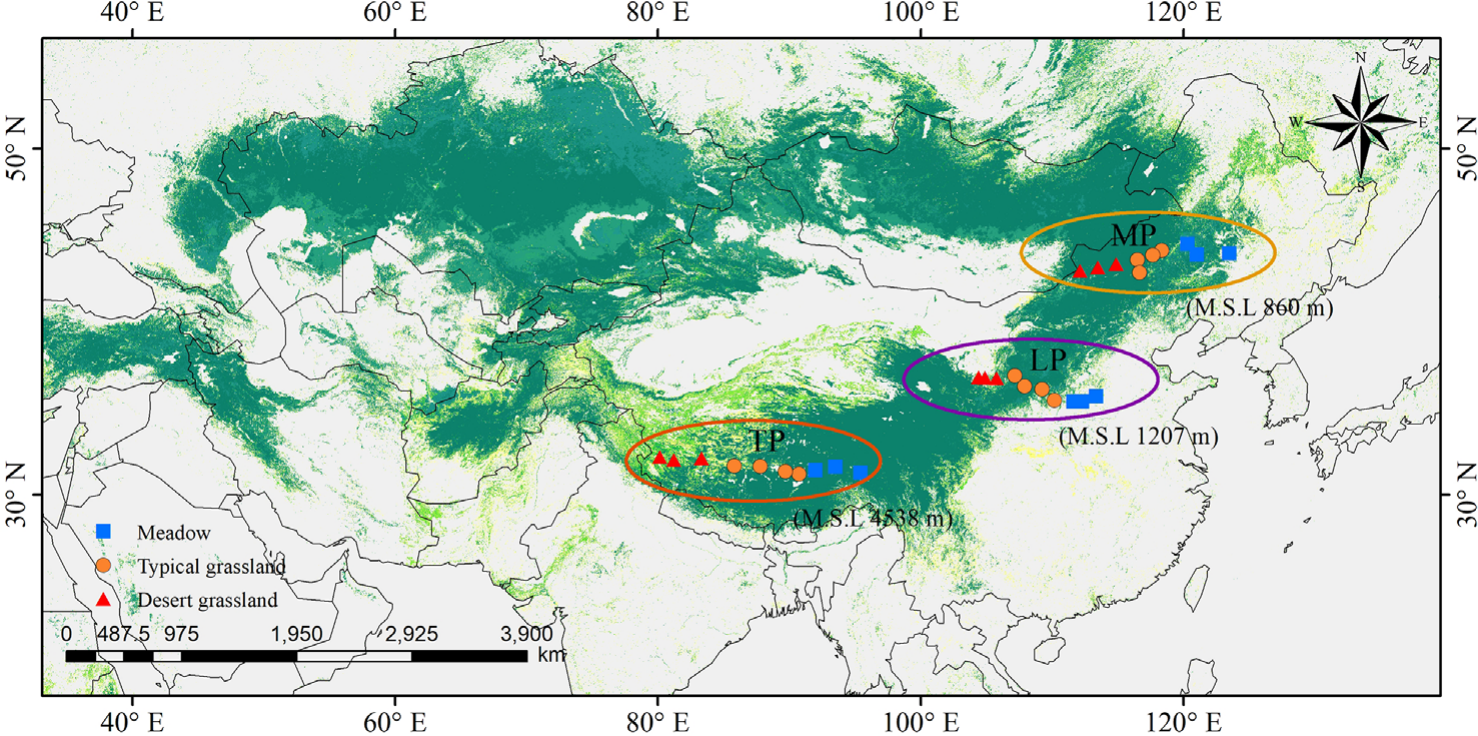
Site locations in northern grassland transects. They were chosen involving three major plateaus in the northern hemisphere, as Mongolia Plateau (MP), Loess Plateau (LP) and Tibet Plateau (TP), and three grassland types of meadow, typical grassland and desert grassland.

Grasslands of the Eurasian continent are representative of Northern hemisphere grasslands. The core part of the Eurasian steppe is mainly distributed in three plateaus: MP, LP, and TP (**Figure 1**). The three plateaus are all characterized by intensive radiation but differ in limiting factors. Specifically, water, soil N, and temperature are limiting in MP, LP, and TP, respectively (**Table 1**). Coincidentally, these limiting factors, as well as excessive radiation, are thought to exert effects on chl. We sampled 30 sites, mainly across precipitation gradients that were not subject to human disturbance in the three plateau grasslands (10 sites per plateau). In addition, the 10 sites in each plateau contained three grassland types: three sites for MD, four sites for TG, and 10 sites for DG (**Figure 1**). Large geographic and climate gradients were also involved in site selection. The longitude range was 80.15–123.51°E, latitude 31.38–45.11°N, altitude 144–4938 m, a mean annual temperature (MAT) −6.76°C to 11.85°C, and mean annual precipitation (MAP) of 140.25–516.55 mm (**Table 1**).

**Table 1:** Geographical locations and environmental conditions of studied sites in northern hemisphere grassland transects.

Site	Region	Grassland type	LAT (°N)	LONG (°E)	ALT (m)	PAR(mol·m^-2^·d^-1^)	MAP(mm)	SNP	MAT (°C)
1	MP	MD	44.59	123.51	144	31.88	448.47	2.49	5.10
2	MP	MD	44.52	121.04	269	33.72	384.39	6.52	5.80
3	MP	MD	45.11	120.33	660	34.47	384.47	5.86	3.72
4	MP	TG	44.77	118.36	1019	35.61	403.58	5.75	0.56
5	MP	TG	44.26	116.52	1129	36.32	315.49	3.63	1.17
6	MP	TG	43.55	116.67	1272	36.05	385.81	3.98	0.16
7	MP	TG	44.51	117.68	1024	35.47	344.68	6.18	1.96
8	MP	DG	44.01	114.89	1101	36.75	281.18	3.85	0.10
9	MP	DG	43.84	113.50	1022	37.09	213.16	2.22	2.47
10	MP	DG	43.63	112.15	955	36.79	168.29	1.57	3.69
11	LP	MD	36.29	113.36	804	28.93	563.88	1.99	11.85
12	LP	MD	35.99	112.29	894	29.70	591.18	3.72	9.96
13	LP	MD	35.99	111.64	833	29.40	566.12	2.73	10.66
14	LP	TG	36.07	110.18	966	32.54	533.36	1.94	10.72
15	LP	TG	36.74	109.24	1268	35.46	498.89	1.60	9.50
16	LP	TG	36.93	107.92	1383	36.21	438.09	1.70	7.46
17	LP	TG	37.58	107.19	1535	37.26	395.14	1.65	5.23
18	LP	DG	37.42	105.78	1293	37.22	320.35	1.37	5.87
19	LP	DG	37.44	104.92	1378	36.81	233.88	1.00	7.56
20	LP	DG	37.46	104.44	1714	36.69	215.50	1.06	7.71
21	TP	MD	31.46	95.45	4104	35.80	619.86	7.74	0.41
22	TP	MD	31.85	93.53	4509	35.70	536.77	5.39	-1.50
23	TP	MD	31.64	92.01	4587	36.77	501.38	6.74	-4.37
24	TP	TG	31.38	90.74	4617	37.53	526.89	3.75	-6.76
25	TP	TG	31.54	89.72	4588	37.24	443.79	4.94	-3.06
26	TP	TG	31.87	87.82	4570	39.01	389.09	5.62	-2.57
27	TP	TG	31.92	85.84	4938	39.77	376.17	2.62	-3.77
28	TP	DG	32.41	83.34	4578	40.21	317.13	1.02	-3.90
29	TP	DG	32.30	81.23	4558	41.13	291.42	0.79	-3.49
30	TP	DG	32.48	80.15	4328	41.92	191.71	1.15	-1.27

MP, Mongolia Plateau; LP, Loess Plateau; TP, Tibet Plateau; MD, meadow; TG, typical grassland; DG, desert grassland; LAT, latitude; LONG, longitude; ALT, altitude; PAR, photosynthetic active radiation; MAP, mean annual precipitation; SNP, the ratio of soil nitrogen to phosphorus; MAT, mean annual temperature.

### Field Sampling

Field sampling was performed in July–August 2018 at the growth peak of the grasslands. In natural communities, the dominant species do not really represent all the species. Hence, at each site, all species appeared within a 1 km radius circle (including trees, shrubs, and herbs) were surveyed and identified. They were thought to be more accurate representatives of all species within this site. Overall, 421 species were collected from 30 sites. The healthy and fully expanded canopy leaves of these trees, shrubs, and herbs were collected, with three individuals used as replicates for each species. The samples were refrigerated in opaque plastic bags until determination. Part of the plant samples was washed and dried in a 60°C oven until a constant weight was reached, used for water content analysis, and then powdered using a grinding machine (MM400 ball mill, Retsch, Haan, Germany) for leaf N measurements. The remaining fresh leaves were used for chl measurements. In addition, soil from the 0–10 cm layer was sampled randomly with eight replicates at each site, naturally air dried, and impurities removed and sieved through a 2-mm mesh. The soil samples were then ground (MM400 ball mill, Retsch, Germany) and stored at 4°C for total N and total phosphorus (P) analysis.

### Measurement of Chl

The Chl measurement followed the principle of Arnon’s simultaneous-equation (Arnon, 1949) and Porra’s improvement (Porra and Scheer, 2019), with a few alterations to improve suitability for the determination of large sample sizes. We used 95% ethanol in place of 80% acetone to avoid the photooxidation of chl and the volatile toxicity of acetone. The ethanol method was put forward in 1981, and the efficiency of 95% ethanol for chl extract was higher than that of acetone (the method is presented at http://www.irgrid.ac.cn/). Owing to the large sample size, we tried our best to operate the same step at the same time of the day, and all the steps were under the dim surrounding light. In detail, the following steps occurred: fresh leaves of samples of 0.1 g were weighed, powdered, and extracted three times using 95% ethanol for chl measurements. The extraction solutions were then combined, filtered, and adjusted to a volume of 50 ml. The measurements were performed using a spectrophotometer (UV-1700, Shimadzu, Japan). Governed by the Beer-Lambert law, the relationships between the chl solution and optical density are (Li et al., 2018b; Li et al., 2018c):

(1) $${\text{D}}_{665}=83.31{\text{ C}}_{\text{a}}+18.60{\text{ C}}_{\text{b}}$$

(2) $${\text{D}}_{649}=24.54{\text{ C}}_{\text{a}}+44.24{\text{ C}}_{\text{b}}$$

where D_665_ and D_649_ were the optical densities of the chl solution at wavelengths of 665 and 649 nm, respectively; C_a_ and C_b_ were chl*a* and chl*b* concentrations (g·L^−1^), respectively; the coefficients 83.31 and 18.60 were the specific absorption of chl*a* and chl*b* at a wavelength of 665 nm, respectively, and 24.54 and 44.24 at 649 nm (Li et al., 2018b; Li et al., 2018c). Chl*a* and chl*b* concentration per gram fresh weight (FW) can be calculated as follows:

(3) $$\text{chl}a\;(\text{mg}\cdot {\text{g}}^{-1}\text{ FW})={\text{C}}_{\text{a}}\times 50/(1000\times 0.1)$$

(4) $$\text{chl}b\;(\text{mg}\cdot {\text{g}}^{-1}\text{ FW})={\text{C}}_{\text{b}}\times 50/(1000\times 0.1)$$

Then, the chl concentration per gram fresh weight (FW) was converted to the reference of dry weight (DW) to deduce the influence of water content:

(5) $$\text{chl}a\;(\text{mg}\cdot {\text{g}}^{-1}\text{ DW})=\text{chl}a\text{ (mg}\cdot {\text{g}}^{-1}\text{FW)/(1-W\%)}$$

(6) $$\text{chl}b\;(\text{mg}\cdot {\text{g}}^{-1}\text{ DW})=\text{chl}b\text{ (mg}\cdot {\text{g}}^{-1}\text{FW)/(1-W\%)}$$

(7) $$\text{total chl}=\text{chl}a\;(\text{mg}\cdot {\text{g}}^{-1}\text{ DW})+\text{chl}b\;(\text{mg}\cdot {\text{g}}^{-1}\text{ DW})$$

(8) $$\text{chl ratio}=\text{chl}a\;(\text{mg}\cdot {\text{g}}^{-1}\text{ DW})/\text{chl}b\;(\text{mg}\cdot {\text{g}}^{-1}\text{ DW})$$

where W% was the percentage of species water content.

### Measurement of Soil N, P, and Leaf N

Soil samples (150 mg) and plant samples (35 g) were used for total N concentration measurements using an elemental analyzer (Vario MAX CN Elemental Analyzer, Elementar, Germany; Zhao et al., 2016a). In addition, 0.05 g soil samples were soaked in a solution of 6 ml of HNO_3_ and 3 ml of HF overnight for P measurements. The solutions were boiled for digestion (Mars X press Microwave Digestion System, CEM Corporation, NC, Matthews, USA), and 0.5 ml of HCLO_4_ was added to remove the acid. Finally, the solutions were cooled and the volumes were adjusted to 15 ml for total P measurements (Zhao et al., 2016b). Determinations were conducted using an inductively coupled plasma optical emission spectrometer (Optima 5300 DV, Perkin Elmer, Waltham, MA, USA). After determination of soil total N and P, the ratio of N:P was calculated. Finally, the averages of soil N:P for each site and the average leaf N for each species were calculated.

### Climate Data

The data for photosynthetic active radiation (PAR) were obtained from “A dataset of reconstructed photosynthetically active radiation in China (1961–2014)” (Liu et al., 2017). Mean annual temperature (MAT) and mean annual precipitation (MAP) for each site were obtained from the National Meteorological Information Center of China.

### Data Analysis

The average was calculated for each species, and all species were used to analyze frequency distributions of chlorophyll concentration and ratio by OriginPro 2018C (OriginLab Corp., Northampton, MA, United States). One-way analysis of variance (ANOVA) was used to test the significance of chl averages among plateaus, grassland types, and functional groups, and multiple comparison tests were performed using the least significant difference method in IBM SPSS Statistics 19.0 (SPSS Inc., Chicago, IL, United States). The linear regressions between species average chl and site average environmental factors were performed using IBM SPSS Statistics 19.0. Standardized major axis (SMA) regressions were used to reveal the relationships of species average leaf N and chlorophyll as well as compare the slopes among groups, performed using the *smart* package in *R* (version 3.5.2, R Foundation for Statistical Computing), and figures were constructed using OriginPro 2018C (OriginLab Corp., Northampton, MA, United States).

## Results

### Distributions and Variations in Leaf Chl

**Figure 2:**
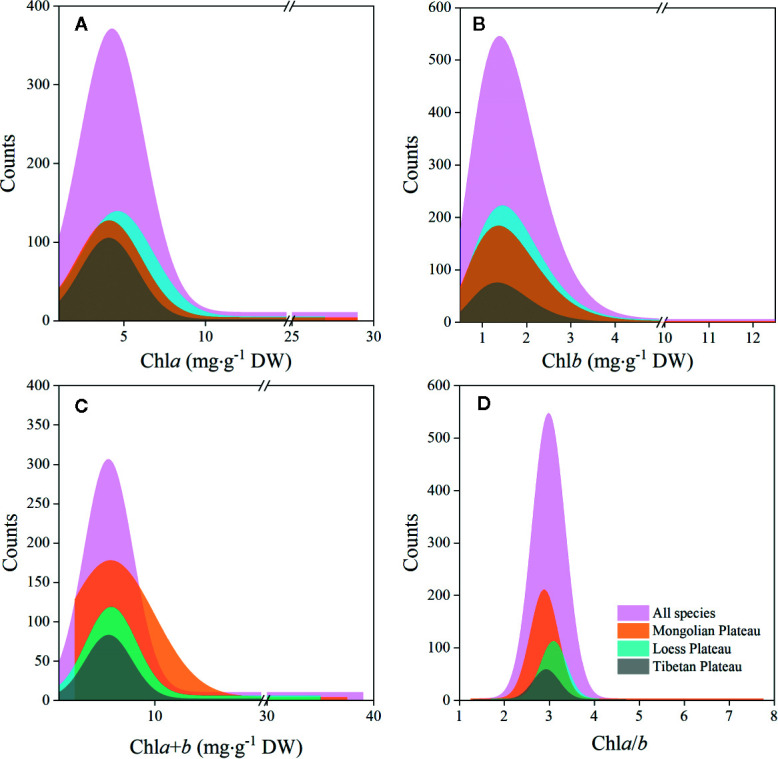
Frequency distributions of chlorophyll concentrations and ratio of the whole northern hemisphere grassland transects and three plateaus, respectively. **(A–D)** denote distributions of chl*a*, chl*b*, chl*a*+*b*, and chl*a*/*b*, respectively.

**Figure 3:**
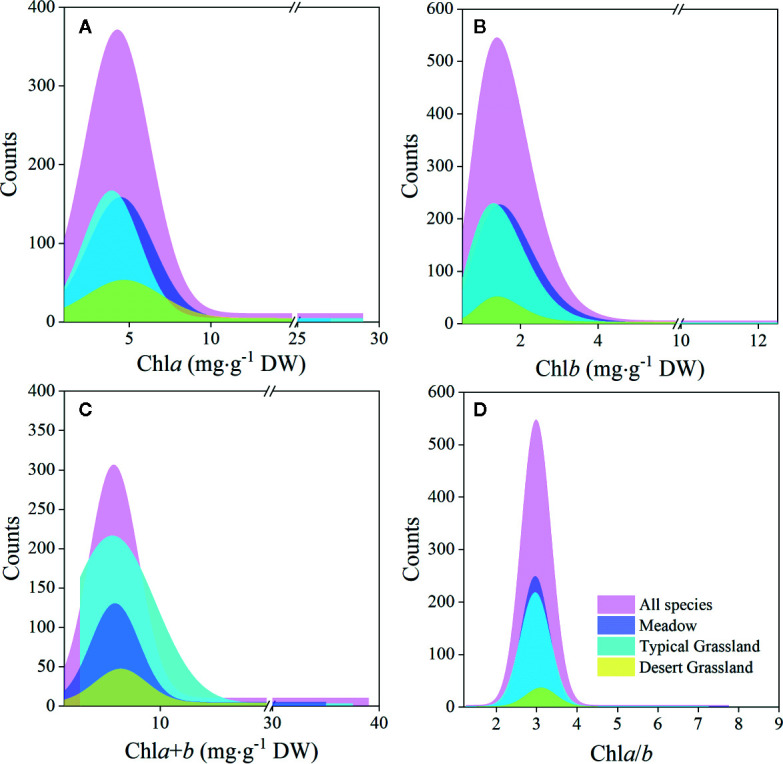
Frequency distributions of chlorophyll concentrations and ratio of the whole northern hemisphere grassland transects and three grassland types, respectively. **(A–D)** denote distributions of chl*a*, chl*b*, chl*a*+*b*, and chl*a*/*b*, respectively.

The chl*a* distribution was in the range of 0.52–28.33, with a mean value of 5.49 mg·g^−1^DW; chl*b* was 0.15–12.11, with a mean value of 1.83 mg·g^−1^ DW; chl*a*+*b* was 0.67–39.29, with a mean value of 7.32 mg·g^−1^ DW; and chl *a*/*b* was 1.28–7.84, with a mean value of 3.02 (**Figures 2** and **3**; **Tables 2** and **3**). Significant positive skewed distributions were found in all grassland transects except chl*a*/*b* of LP, TP, and DG (**Tables 2** and **3**). In addition, the kurtosis of chl concentrations (chl*a*, chl*b*, and chl*a*+*b*) and ratio (chl*a*/*b*) in DG were far lower compared with other groups (**Table 3**).

**Table 2:** Eigen-values of frequency distributions of chlorophyll concentrations by dry weight (DW) and ratio grouped by regions.

	Chl*a* (mg·g^-1^ DW)				Chl*b* (mg·g^-1^ DW)				Chl*a*+*b* (mg·g^-1^ DW)				Chl*a*/*b*			
	All*	MP*	LP*	TP*	All*	MP*	LP*	TP*	All*	MP*	LP*	TP*	All*	MP*	LP	TP
**Mean**	5.49	5.55**a**	5.90**a**	4.65**b**	1.83	1.87**a**	1.91**a**	1.63**b**	7.32	7.42**a**	7.81**a**	6.29**b**	3.02	3.01**b**	3.11**a**	2.88**c**
**SD**	3.35	3.79	3.34	2.41	1.13	1.32	1.10	0.84	4.46	5.06	4.42	3.22	0.49	0.63	0.34	0.42
**Variance**	11.24	14.40	11.13	5.80	1.29	1.73	1.22	0.70	19.85	25.64	19.55	10.36	0.24	0.40	0.12	0.17
**CV**	0.61	0.68	0.57	0.52	0.62	0.71	0.58	0.52	0.61	0.68	0.57	0.51	0.16	0.21	0.11	0.15
**Min**	0.52	0.78	0.52	0.59	0.15	0.16	0.15	0.22	0.67	1.25	0.67	0.81	1.28	1.28	1.81	1.42
**Max**	28.33	28.33	26.19	17.95	12.11	12.11	9.43	6.22	39.29	39.29	35.62	23.73	7.84	7.84	4.14	4.57
**Median**	4.61	4.46	5.06	4.29	1.55	1.55	1.63	1.48	6.15	5.97	6.71	5.81	3.00	2.90	3.10	2.91
**Skewness**	2.27	2.61	1.68	2.10	2.90	3.45	2.03	2.07	2.39	2.77	1.76	2.11	2.42	3.29	0.04	-0.02
**Kurtosis**	8.40	9.94	4.60	7.53	15.56	18.87	7.24	7.43	9.70	11.56	5.20	7.65	20.02	19.44	1.03	2.11

All, all species along northern hemisphere grassland transects (n=1049); MP, Mongolia Plateau (n=367); LP, Loess Plateau (n=437); TP, Tibet Plateau (n=245). The asterisks behind the abbreviation of regions denote significant differences with normal distributions. Bold lowercase letters behind means denote significant differences among regions (p < 0.05).

**Table 3:** Eigen-values of frequency distributions of chlorophyll concentrations by dry weight (DW) and ratio grouped by grassland types.

	Chl*a* (mg·g^-1^ DW)				Chl*b* (mg·g^-1^ DW)				Chl*a*+*b* (mg·g^-1^ DW)				Chl*a*/*b*			
	All*	MD*	TG*	DG*	All*	MD*	TG*	DG*	All*	MD*	TG*	DG*	All*	MD*	TG*	DG
**Mean**	5.49	5.58**b**	5.06**c**	6.23**a**	1.83	1.88**a**	1.71**b**	2.00**a**	7.32	7.46**b**	6.77**c**	8.24**a**	3.02	3.01**b**	2.98**b**	3.12**a**
**SD**	3.35	3.42	3.10	3.59	1.13	1.17	1.09	1.13	4.46	4.56	4.16	4.69	0.49	0.53	0.45	0.43
**Variance**	11.24	11.73	9.64	12.88	1.29	1.37	1.18	1.27	19.85	20.79	17.34	21.99	0.24	0.28	0.20	0.19
**CV**	0.61	0.61	0.61	0.58	0.62	0.62	0.64	0.57	0.61	0.61	0.61	0.57	0.16	0.18	0.15	0.14
**Min**	0.52	0.59	0.94	0.52	0.15	0.22	0.16	0.15	0.67	0.81	1.37	0.67	1.28	1.28	1.42	1.53
**Max**	28.33	26.19	28.33	18.77	12.11	9.76	12.11	6.14	39.29	35.62	39.29	24.53	7.84	7.84	7.35	4.39
**Median**	4.61	4.71	4.29	5.30	1.55	1.62	1.45	1.75	6.15	6.28	5.76	6.98	3.00	2.98	2.98	3.11
**Skewness**	2.27	2.43	2.69	1.31	2.90	2.84	3.87	1.27	2.39	2.50	2.96	1.30	2.42	3.27	2.21	-0.21
**Kurtosis**	8.40	8.99	13.62	1.62	15.56	13.01	28.06	1.44	9.70	9.65	16.65	1.54	20.02	24.40	21.19	0.91

All, all species along northern hemisphere grassland transects (n=1049); MD, meadow (n=444); TG, typical grassland (n=421); DG, desert grassland (n=184). The asterisks behind the abbreviation of regions denote significant differences with normal distributions. Bold lowercase letters behind means denote significant differences among grassland types (p < 0.05).

Significant differences in the means of chl concentration and ratio were observed among the plateaus, and were higher in MP and LP, and lower in TP (**Table 2**). For different grassland types, the chl concentration and ratio of DG were significantly higher, and TG was lower than in the other groups (**Table 3**).

Regardless of region and grassland type, the coefficients of variation (CVs) of chl concentration were all approximately 0.6, whereas the CVs of chl*a*/*b* were less than half of those of concentration (**Tables 2** and **3**). Among the plateaus, the chl concentration CV ranking was MP > LP > TP, and the Chl ratio CV ranking was MP > TP > LP (**Table 2**). Among grassland types, DG had the lowest CV for chl concentrations and chl*a*/*b* (**Table 3**).

### Variations in Leaf Chl Among Different Functional Groups

For chl concentration and ratio, significant differences existed among plant life forms, life spans, and taxonomies (**Table 4**): for different life forms, different chl*b* (i.e., highest for herbs and lowest for trees) resulted in lower chl*a*/*b* for herbs; for different life spans, the lowest chl*a*, chl*b*, and chl*a*+*b* were found for perennials and the highest chl*a*/*b* for annuals; for different taxonomies, chl*a*, chl*b*, and chl*a*+*b* of Gramineae were significantly lower, whereas chl*a*/*b* of Compositae was significantly lower; also, significant differences in chl existed between monocotyledons and dicotyledons, with higher chl concentration and lower chl*a*/*b* for dicotyledons; as the dominant and common species, the chl concentrations were lower than in other species, but the chl*a*/*b* were similar to other species.

**Table 4:** One-way ANOVA for chl*a*, chl*b*, chl*a*+*b* concentration by dry weight (DW) and chl*a*/*b* of different functional groups in northern hemisphere grassland transects.

Functional groups	Chl*a* (mg·g^−1^)	Chl*b* (mg·g^−1^)	Chl*a*+*b* (mg·g^−1^)	Chl*a*/*b*
	Mean ± sd	Mean ± sd	Mean ± sd	Mean ± sd
**Trees** (80)	5.06 ± 2.54	1.57 ± 0.75**b**	6.63 ± 3.29	3.20 ± 0.31**a**
**Shrubs** (101)	5.20 ± 3.71	1.7 ± 1.3**ab**	6.91 ± 4.97	3.11 ± 0.56**a**
**Herbs** (862)	5.56 ± 3.38	1.87 ± 1.15**a**	7.43 ± 4.49	2.99 ± 0.48**b**
**Annuals** (137)	7.23 ± 3.79**a**	2.28 ± 1.20**a**	9.51 ± 4.95**a**	3.17± 0.56**a**
**Biennials** (56)	6.85 ± 4.44**a**	2.42 ± 1.83**a**	9.27 ± 6.25**a**	2.90 ± 0.27**b**
**Perennials** (760)	5.02 ± 2.83**b**	1.68 ± 0.95**b**	6.71 ± 3.76**b**	3.01 ± 0.46**b**
**Legumes** (119)	5.39 ± 2.71**ab**	1.85 ± 0.95**a**	7.24 ± 3.64**ab**	3.02 ± 0.61**a**
**Gramineae** (158)	4.96 ± 2.77**b**	1.57 ± 0.77**b**	6.53 ± 3.52**b**	3.11 ± 0.36**a**
**Compositae** (169)	5.77 ± 3.34**a**	2.00 ± 1.14**a**	7.77 ± 4.47**a**	2.88 ± 0.26**b**
**Others** (601)	5.53 ± 3.47**ab**	1.84 ± 1.19**a**	7.37 ± 4.62**a**	3.04 ± 0.52**a**
**Monocotyledon** (234)	4.99 ± 3.10**b**	1.61 ± 1.00**b**	6.60 ± 4.07**b**	3.09 ± 0.34**a**
**Dicotyledon** (796)	5.64 ± 3.32**a**	1.90 ± 1.13**a**	7.53 ± 4.41**a**	3.00 ± 0.52**b**
**Dominant species** (78)	4.70 ± 2.13**b**	1.53 ± 0.68**b**	6.23 ± 2.77**b**	3.07 ± 0.36
**Common species** (196)	5.15 ± 3.11**ab**	1.70 ± 0.89**b**	6.85 ± 3.94**ab**	3.01 ± 0.52
**Other species** (773)	5.63 ± 3.40**a**	1.88 ± 1.18**a**	7.51 ± 4.56**a**	3.02 ± 0.49

Mean ± standard deviations are shown and numbers of samples (n) in parentheses. Bold lowercase letters denote significant differences among groups (p < 0.05).

### Factors Influencing Leaf Chl

**Figure 4:**
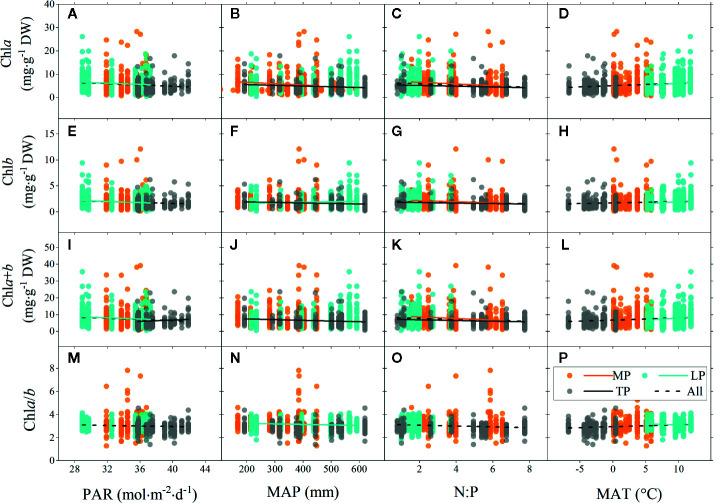
Linear regressions of chlorophyll concentrations (chl*a*, chl*b*, and chl*a*+*b*) and ratio (chl*a*/*b*) to major environmental factors grouped by regions. **(A–D)**, **(E–H)**, **(I–L)**, and **(M–P)** denote regressions of chla, chlb, chla+b, and chla/b to four environmental factors, respectively. MP, Mongolia Plateau; LP, Loess Plateau; TP, Tibet Plateau; PAR, photosynthetic active radiation; MAP, mean annual precipitation; N:P, the ratio of soil nitrogen to soil phosphorus; MAT, mean annual temperature. Lines denote significant linear fitted relationships (*p* < 0.05).

Significant linear relationships were observed between chl and major environmental factors over the entire grassland (**Figure 4**). The chl concentrations (chl*a*, chl*b*, and chl*a*+*b*) were negatively correlated to PAR and N:P, but positively correlated to MAT over the entire grassland. Similar trends were also observed for chl ratio. However, there was no linear relationship between chl and MAP, in either the concentration or the ratio, for the entire grassland (**Figures 4B, F, J, N** **)**.

Among the three plateau regions, some differences in chl were observed for different environments. The chl concentration of MP was mainly negatively correlated to N:P (**Figures 4C, G, K** **)**. The situation of LP was very similar to that of the entire grassland (**Figure 4**). However, unlike for the entire grassland, there was no relationship between chl concentration and N:P in LP (**Figures 4C, G, K** **)**. In addition, chl*a*/*b* of LP was negatively correlated to MAP (**Figure 4N**) but positively correlated to MAT (**Figure 4P**). Chl concentration in TP was mainly negatively affected by MAP and N:P.

**Figure 5:**
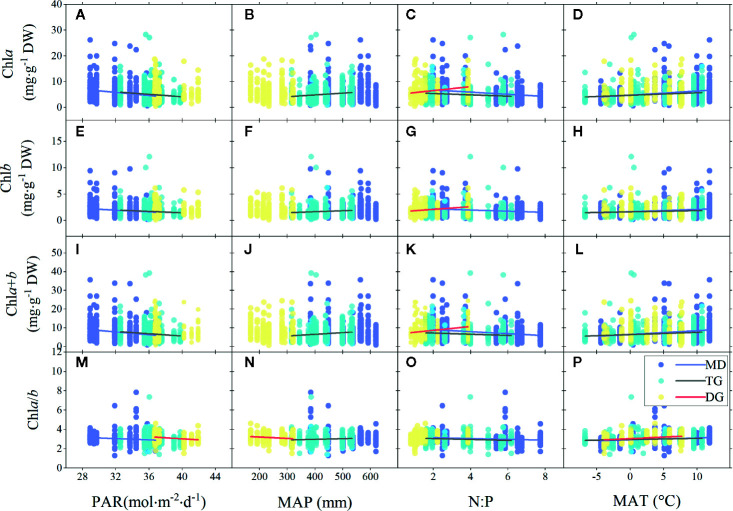
Linear regressions of chlorophyll concentrations (chl*a*, chl*b*, and chl*a*+*b*) and ratio (chl*a*/*b*) with major environmental factors grouped by grassland types. **(A–D)**, **(E–H)**, **(I–L)**, and **(M–P)** denote regressions of chla, chlb, chla+b, and chla/b to four environmental factors, respectively. MD, meadow; TG, typical grassland; DG, desert grassland; PAR, photosynthetic active radiation; MAP, mean annual precipitation; N:P, ratio of soil nitrogen to soil phosphorus; MAT, mean annual temperature. Lines denote significant linear fitted relationships (*p* < 0.05).

For different grassland types, the chl concentration trends across the MD and TG environments were the same as for the whole grasslands (**Figures 5A–P**), but chl (concentration and ratio) of TG was also positively related to MAP (**Figures 5B, F, J, N** **)**. In DG, decoupling was found between chl concentration and most environmental factors, except for positive relationships with soil N:P (**Figures 5C, G, K** **)**, and a decrease in chl ratio decreased with increasing PAR and MAP, but increased with MAT (**Figures 5M, N, P** **)**.

### Allometric Allocations of Leaf N to Leaf Chl

**Figure 6:**
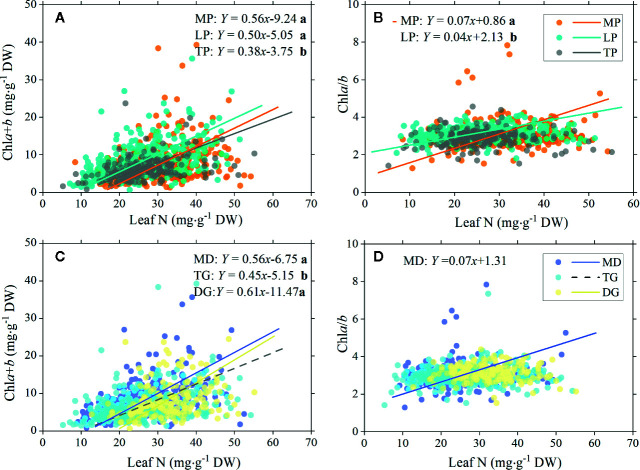
Standardized major axis (SMA) regression of chlorophyll total concentration (chl*a*+*b*) and ratio (chl*a*/*b*) to leaf N for different regions and grassland types. Relationships between total chlorophyll concentration and leaf nitrogen **(A)**, and between chlorophyll ratio and leaf nitrogen **(B)** for three plateau regions are showed , respectively. Corresponding relationships between chlorophyll and leaf nitrogen in three grassland types are showed in **(C, D)**. Lines denote significant linear relationships (*p* < 0.05). Fitting functions are given only for significant linear relationships. Different lowercase letters behind functions indicate significant difference between SMA slopes (*p* < 0.05). MP, Mongolia Plateau; LP, Loess Plateau; TP, Tibet Plateau; MD, meadow; TG, typical grassland; DG, desert grassland.

There were significant positive relationships between total chl concentration and leaf N in MP, LP, and TP, but the SMA slopes were significantly different, that is, higher in MP and LP (0.56 and 0.50, respectively) and lower in TP (0.38; **Figure 6A**). Between chl*a*/*b* and leaf N, positive linear relationships were found in MP and LP, with a significantly higher slope for MP (**Figure 6B**).

For all three types of grassland, significant positive linear relationships between total chl concentration and leaf N were found, but with a significantly lower slope for TG (**Figure 6C**). MD showed a significant relationship between chl*a*/*b* and leaf N (**Figure 6D**).

## Discussion

### Chl Distributions in the Northern Hemispheric Grasslands

The values for chl*a*, chl*b*, chl*a*+*b*, and chl*a*/*b* ranged within 0.52–28.33, 0.15–12.11, and 0.67–39.29 mg·g^−1^ DW, and 1.28–7.84, respectively, across 421 species in these grasslands (**Figures 2** and **3**; **Tables 2** and **3**). Positive skewness, regardless of region or grassland type (except for chl*a*/*b* of TP and DG, which exhibited non-significant skewed distributions), showed that the majority of species converged at lower chl concentrations and ratios. According to a previous study on tropical to temperate forests, the total chl concentration ranged from 1.20–22.58 mg·g^−1^ FW, showing grater plasticity compared with grasslands when converted to dry weight (Li et al., 2018b). The large plasticity is due to adaptation to large light intensity and quality variations from canopy to understory within forest communities (Kitajima and Hogan, 2003; Hallik et al., 2009). In contrast, excessive radiation in plateau grasslands may be the reason for the lower chl concentration, because acclimation to high light can result in chloroplast reduction activated by photo-protection (Hallik et al., 2009). Furthermore, grasslands are generally characterized by a relatively simpler layer structure compared with that of forests, allowing nearly constant light intensity and quality for species to converge (Terashima and Hikosaka, 1995; Valladares and Niinemets, 2008; Bachmann et al., 2018). However, conservative chl*a*/*b* was found in forests (1.43–7.07; Li et al., 2018b) and grasslands (1.28–7.84) due to the linear relationships between chl*a* and chl*b*, which may indicate a fixed chl composition in land plants, regardless of biome and vegetation type.

There were significant differences in the mean chl concentration and ratio among the three plateaus: higher in MP and LP and lower in TP. The environmental N supply plays a key role in Chl concentration (Fajardo and Piper, 2017). Soil N is the primary limiting factor for plant growth in LP (Yu et al., 2019), and plants may have to maximize productivity by allocating more N to chl and Rubisco. It has been reported that under high-light conditions, N limitation may affect partitioning between Rubisco and photosynthetic apparatus (Kitajima and Hogan, 2003). Consistently, we found that greater amounts of leaf N were invested in chl and resulted in higher chl in the N-limited LP (**Figure 6A**, **Table 2**). In TP, even if N was sufficient to form chl, the plants’ production rate would still be limited by low temperatures (**Table 1**). Simultaneously, plants must enhance photoprotective capacity by downregulating photosynthesis and upregulating photorespiration under intensive direct radiation (Zhang et al., 2019). Under these conditions, reducing N allocation to chl would be cost-effective for plants. In addition, the formation of protective and resistant proteins is needed under cold and high-light conditions to sustain survival (Fajardo and Piper, 2017), and there will also be a competitive N pool constraining chl synthesis. The physiological synergy of these pathways results in lower investment in chl for TP compared with the other regions (**Figure 6A**, **Table 2**).

The highest chl*a*, chl*b*, chl*a*+*b*, and chl *a*/*b* were found in DG compared with other grassland types (**Table 3**). The vegetation in DG is sparse and exposed to direct sunlight (i.e., strong direct radiation); therefore, species tend to increase chl concentration and chl *a*/*b* to improve direct radiation (red-orange light) use efficiency (Hansen et al., 2002; Valladares and Niinemets, 2008). In addition, the lower kurtosis in DG implies a relative higher trait diversity (Gross et al., 2017). The desert is a long-term stressful ecosystem; hence, species will evolve a series of strategies (such as tolerance or avoidance) to successfully adapt and survive (Schamp et al., 2008; Gross et al., 2009). This may also produce diverse trait phenotypes. Unexpectedly, it was observed that plants in desert ecosystems implement multiple functions by producing diverse trait phenotypes (Gross et al., 2017).

### Chlorophyll Variations Depending on Functional Groups in the Northern Hemisphere Grasslands

The evolution of leaf traits is usually influenced by plant growth form, environment, and taxonomy (Stock and Verboom, 2012; Zhao et al., 2014; Wang et al., 2015). In our study, chl differences mainly existed among life forms, life spans, and taxonomies (**Table 4**). Only chl*b* increased significantly in trees, shrubs, and herbs, resulting in a significant decrease in chl*a*/*b* from trees to herbs. This result was consistent with the results from forests, which reflect the adaptation to environmental light produced by the layer structure (Li et al., 2018b). Higher chl*b* not only represents larger antennae size to achieve efficient use of diffused light, but its protein complex (LHCII) also mediates energy distribution between PSI and PSII over variable photon flux densities; this is favorable for herbs in plateaus with excessive radiation energy (Andrews et al., 1995; Grieco et al., 2015).

Compared with perennials, the chl concentrations in annuals and biennials were significantly higher (**Table 4**). According to the leaf economics spectrum, a long life span requires robustness and persistence, whereas a short life span requires quick return on investment of nutrients (Wright et al., 2004). That is, product-related traits, such as leaf N and leaf P, and the photosynthetic rate of annuals and biennials would be higher than that of perennials. As the initiators of plant production, higher chl concentrations are the basis of quick metabolism and growth in the short life span of annuals and biennials.

There were also chl differences among different taxonomies. Gramineae had the lowest chl concentrations and Compositae had the lowest chl*a*/*b* compared with other families (**Table 4**). This may be related to the functional and strategic differences among taxa. Gramineae are the most dominant in grasslands and are generally characterized by efficient resource utilization (Emilie et al., 2015). A small amount of chl produces higher biomass for Gramineae, which is a reflection of the high efficiency of light use. Similarly, the chl concentration of dominant species was lower than that of other species. Compositae were mainly distributed in cold alpine areas and increased Rubisco activity at the cost of decreased chl*a*/*b* can help these species cope with possible damage from cold and strong radiation, as well as sustaining production (Castrillo, 2006). The chl differences between classes (**Table 4**) also resulted from the differences among families because Gramineae are monocotyledons, whereas Fabaceae, and Compositae are dicotyledons. According to other studies, species-specific light acclimation is commonly due to different habitat distributions (Murchie and Horton, 1997; Hansen et al., 2002). For other leaf traits in the Chinese grasslands of Inner Mongolia, Tibet, and Xinjiang, on average, 27% of the total variation was due to taxonomy or phylogeny (He et al., 2010; Liu et al., 2013). This suggests that the common effects of environmental and biotic drivers may drive chl evolution; however, more evidence is needed.

### Factors Influencing Leaf Chl in the Northern Hemisphere Grasslands

Radiation is one of the most critical factors determining chl concentrations. For woody species, chl concentration generally decreases when light is more available. Although this was confirmed partly by our results in grasslands, different mechanisms may be involved (Murchie and Horton, 1997; Hansen et al., 2002). Acclimation to light may occur through multiple mechanisms in the leaf or chloroplast, physiologically or morphologically, such as, adjustment of chlorophyll concentration, chloroplast numbers, specific leaf area, and mesophyll layers, as well as activating photo-protection and balancing efficiency of different photosynthetic apparatus (Kull, 2002; Hallik et al., 2009). Contrasting canopy structures of forest and grassland may influence the dominance of these factors to produce different light adaptation results (Hallik et al., 2009).

As biochemical reactions, the synthesis and degradation of chl can also be influenced by the surrounding water, nutrients, and temperature as well as their interactions. As generally acknowledged, regions of higher precipitation and temperature with fertile soil will result in higher productivity (Knapp and Smith, 2001; Bai et al., 2008; Huang et al., 2017). Instead, in the current study, for the plateau regions, chl concentration was lower in wetter and fertile places (**Figure 4**). First, the amount of precipitation may not be the determining factor that linearly affects soil water and nutrient availability, though the three interactions in most cases. Some studies documented that soil N availability was high in moderately humid areas but was limited in areas that are too dry or too wet owing to drought stress and leaching loss (Sitaula and Bakken, 1993; Pérez and Armesto, 2017). In addition, the variation in soil water content utilized by plants depended on the pattern of rainfall events, rather than the amount of rainfall, which caused the productivity to be insensitive to the precipitation amount in grasslands (Knapp et al., 2002). In the results, TG was the only area to show positive relationships between chl concentration and MAP, which may indicate the coordinated variations between MAP, soil water, and nutrients. Second, it is also related to the different water storage strategies of species coping with variable MAP: when converting the chl concentration by reference to fresh weight, the relationships with MAP turned out to be positive across all grasslands and in LP. The chl concentration in DG showed positive relationships (**Figures 5C, G, K** **)** with soil N:P, indicating the excellent capacity of desert species in maintaining higher leaf N to overcome production limitations caused by drought stress (Liu et al., 2010; Luo et al., 2015). In summary, the effects of MAP and soil nutrient on chl vary depending on the inherent soil humidity, fertility, and species’ strategy for water and nutrients using, and these interactions make the relationships debatable in regions and across the entire grasslands.

The absence of relationships between chl*a*/*b* and environments and small CV of chl*a*/*b* despite regions or grassland types (**Tables 2** and **3**) indicated that chl*a*/*b* was a relatively stable trait in this grassland. In our study, the chl*a* amount was approximately three times that of chl*b*, as seen for species in the forests (Li et al., 2018b). It is well established that chl*b* evolved to replace phycobiliproteins owing to its “cheaper” bio-energetic cost and space saving (Larkum et al., 2018). This cost-effective amounts of chl*a* and chl*b* was confirmed to show some phylogenetic signals and hence a relative stable trait along with evolution (Li et al., 2018b). However, the trends for the entire grasslands were that chl*a*/*b* decreased in the higher PAR and higher N:P places, but increased in the warmer places. This was in contrast with the general conclusion that chl*a*/*b* increased under high light compared with shade because, chl*b* increases caused by the overexpression of chlorophyllide *a* oxygenase (the precursor for the biosynthesis of chl*b*) under shade to enlarge the antennae size for light harvesting and to enhance electron transport in PSI and PS II (Ajaya et al., 2012; Shao et al., 2014). However, alternative mechanisms regarding interactions of PSI, PS II and LHC were proposed to explain variable chl*a*/*b* under shade, including shifted chl composition in PSI (Ben-Shem et al., 2004), larger size of LHCI (Tan et al., 1995), and increased PSI/PS II ratio (Vladimir et al., 2016; Schwarz et al., 2018). Rather, some studies stated that there was a low difference in chl*a*/*b* between species under sun and shade because the adjustment mainly occurs in connectivity alternation between PSI and PSII (Wyka et al., 2008; Zivcak et al., 2014), or due to the differences in species-specific strategies of shade tolerance and sensitivity (Sekhar et al., 2019). Considering the single layer and excessive radiation of plateau grassland, it may not be necessary to increase the antennae size to harvest photons for species; thus, other mechanisms, depending on the species strategy, age, or environmental threshold, should be taken into account.

### Allocation of Leaf N to Chl in Grasslands

Leaf N is acknowledged as a product-related trait for plants and is used as a proxy for productivity (Angela et al., 2014). It is believed that higher leaf N implies efficient photosynthesis and higher productivity (Reich et al., 1994). In addition, a close link between leaf chl concentration and leaf N content was found. The reason was thought to be that the majority of leaf N was contained in chl molecules (Boussadia et al., 2011). However, during photosynthesis, leaf N may be allocated to one of many competing mechanisms, specifically, to 1) chl and its associated proteins in LHC for light harvesting; 2) chl-protein complexes in RC for electron transport; and 3) the synthesis of photosynthetic enzymes in the Calvin cycle (such as Rubisco and other soluble functional proteins). The allocation of leaf N to these different processes reflects the manner in which the plants implement functional balance to achieve optimal production. In this sense, leaf N–chl relationships reflect N allocation to pigments and the related thylakoid reactions. Owing to the unique role of chl*a* and chl*b* in the RC and LHC, respectively, the leaf N–chl*a*/*b* relationship roughly reflects N allocation between RC and LHC, and also reflects how the efficiency of light harvesting and electron transport is coordinated. In our study, we verified these relationships in grasslands and found different leaf N allocation patterns among regions and grassland types.

First, there were significant positive relationships between leaf N and chl in three plateaus, with higher SMA slopes (0.56 and 0.50) for MP and LP, and a lower slope for TP (**Figure 6A**), denoting the lower leaf N allocated to chl in TP. As a result, chl concentrations in TP were significantly lower than in other regions (**Table 2**). The reduction in N allocation to chl should be cost-effective for plants living in harsh habitats to balance production and persistence. Protection and resistance proteins are needed under cold and high-light conditions in order to sustain survival, and the formation of these functional proteins indeed competes for large amounts of leaf N (Fajardo and Piper, 2017). Second, in TG (the driest), the leaf N that was invested in chl was as much as in MD (the wettest) and resulted in the highest chl total concentration in these grasslands (**Figure 6C**). This was also confirmed by the positive relationships between chl concentration and soil N:P, which reflects a rather efficient utilization of soil N in the DG (**Figures 5C, G, K** **)**. In drought-affected locations, large roots facilitate the exploration of available water and nutrients. It has been shown that morphological adjustments (large root-to-leaf ratio) are the major mechanisms applied for maintaining constant high physiological activities such as photosynthesis in desert plants (Liu et al., 2010; Xie et al., 2015).

The chl*a*/*b* ratio can be a useful indicator of N allocation within the photosynthetic apparatus because it should be positively correlated to the ratio of RC and LHC. Rather, the PS core is more costly than the LHC, and plants generally tend to maintain chl*a*/*b* as low as possible at the same time to achieve a high photosynthesis rate (Larkum et al., 2018). However, under N-limitation and high light, more N will be allocated to RC to improve the maximum photosynthesis rate at the cost of reducing Rubisco and LHC (Kitajima and Hogan, 2003). The increased excitation pressure under high light and N-deficiency is probably the result of saturation of the electron transport chain due to the limitation of the use of reductants by the Calvin cycle. Thus, it is wise to decrease the light-harvesting capacity and increase the thermal dissipation of absorbed energy (Huang et al., 2004). However, we found reductions in chl*a*/*b* with decreased leaf N in MP, LP, and MD (**Figures 6B, D**), which means that leaf N allocates a higher proportion to light harvesting compared with RC under decreased leaf N. It was concluded that LHCII was important for balancing the distribution of excitation energy between PSI and PS II over a wide range of photon flux densities (Andrews et al., 1995), and that chl*b*-deficient wheat mutants severely impacted the photoprotective response (Brestic et al., 2015). In plateau grassland, species must cope with constant excessive radiation throughout their lives, and an excellent capacity for energy dissipation and photoprotection will contribute to PS core efficiency. It is hard to say if these mechanisms are involved in positive chl*a*/*b*–leaf N relationships in our study, as many species living in large environmental gradients. Despite generalities in the acclimation of the N allocation between photosynthetic apparatus for some cases, differences may result from the relative dominance of scaling, environmental, taxonomic, and phylogenetic influences.

There is no doubt that leaf N plays crucial roles in photosynthesis, but other non-photosynthetic metabolic activities such as respiration, defense, and tolerance also compete for leaf N to synthesize functional proteins, especially for species living in harsh environments. The decoupling of chl*a*/*b*–leaf N relationships in TP (temperature stress), TG, and DG (water stress) may be related to the intensive competition for leaf N. It was established that environmental stress intensifies the imbalance between light harvesting and energy dissipation under high light and results in excess PSII excitation pressure. Consequently, the species increase RuBP-regeneration, thus increasing the electron flux through the Calvin cycle (Ivanov et al., 2012). Moreover, as the central component in the nonradiative energy dissipation process, whether carotenoid-related protein complexes also have a high N cost should be investigated in these stressful grasslands.

In summary, different N allocation patterns to photosynthesis and non-photosynthesis among plants from different biomes may cause decoupling of leaf N with productivity, so generalizations relating leaf N to productivity should be made carefully in some cases (Croft et al., 2017). Furthermore, although chl is considered a better predictor of productivity than leaf N in recent years, most of the energy chl absorbed is actually dissipated, and only a small amount is used in the Calvin cycle. Consequently, compared with using a single trait, the allocation of leaf N to photosynthesis or non-photosynthesis, to thylakoid reactions or to the Calvin cycle, to LHC or RC, will provide informative parameters that indicate the functional balance, thereby improving the technology of productivity prediction.

## Conclusions

We reported the distributions, variations, and influencing factors of chl concentrations and ratio in northern hemisphere grassland transects that are involved three distinct plateaus and three grassland types. We concluded that, first, lower distributions and narrower ranges of Chl in grasslands resulted from the single canopy structure of grassland and the constant strong radiation in the plateaus. However, there were differences in the average chl among regions and grassland types, which were due to different adaptations of species under distinct stressful environments. Second, chl varied among life forms, life spans, and taxonomies, implying different light use strategies shaped by both environment and phylogeny. Third, chl concentrations and the chl*a*/*b* ratio were generally negatively related to PAR and N:P, but positively related to MAT in these grasslands. Finally, we found that the allocation patterns of leaf N to chl differed among regions and grassland types, and non-photosynthetic metabolism may compete for leaf N and cause these differences. Thus, in these cases, caution is needed when relating single leaf N or chl to productivity separately.

## Data Availability Statement

The datasets generated for this study are available on request to the corresponding author.

## Author Contributions

NH and GY conceived the ideas and designed methodology. YZ, YL, RW, LX, ZL, ZW, and JZ participated in the field works. YZ, YL, and RW determined the chlorophyll indicators and analyzed the data. YZ, ML, and NH led the writing of the manuscript. All authors contributed to the article and approved the submitted version.

## Funding

The study was supported by National Key R&D Project of China (2017YFA0604803) and Youth Program of National Natural Science Foundation of China (41701053).

## Conflict of Interest

The authors declare that the research was conducted in the absence of any commercial or financial relationships that could be construed as a potential conflict of interest.

## References

[B1] Ajaya K. B. Gopal K. P. Shiv S. Pandey S. L. Vanga S. R. Govindjee (2012). Light intensity-dependent modulation of chlorophyll b biosynthesis and photosynthesis by overexpression of chlorophyllide a oxygenase in tobacco. Plant Physiol. 159, 433–449. 10.1104/pp.112.195859 22419827 PMC3375976

[B2] Andrews J. R. Fryer M. J. Baker N. R. (1995). Consequences of LHC II deficiency for photosynthetic regulation in chlorina mutants of barley. Photosyn. Res. 44, 81–91. 10.1007/BF00018299 24307028

[B3] Angela T. M. Sarah E. P. Shawn W. L. Habacuc F. Monica A. Marianne L. T. (2014). Which is a better predictor of plant traits: temperature or precipitation? J. Veg. Sci. 25, 1167–1180. 10.1111/jvs.12190

[B4] Arnon D. I. (1949). Copper enzymes in isolated chloroplasts. Polyphenoloxidase in *Beta vulgaris* . Plant Physiol. 24, 1–15. 10.1104/pp.24.1.1 16654194 PMC437905

[B5] Atsushi K. Tomoko A. Nishida N. K. (2018). Why is chlorophyll b only used in light-harvesting systems? J. Plant Res. 131, 961–972. 10.1007/s10265-018-1052-7 29992395 PMC6459968

[B6] Bachmann D. Roscher C. Buchmann N. (2018). How do leaf trait values change spatially and temporally with light availability in a grassland diversity experiment? Oikos 127, 935–948. 10.1111/oik.04533

[B7] Bai Y. Wu J. Xing Q. Pan Q. Huang J. Yang D. (2008). Primary production and rain use efficiency across a precipitation gradient on the Mongolia Plateau. Ecology 89, 2140–2153. 10.1890/07-0992.1 18724724

[B8] Ben-Shem A. Frolow F. Nelson N. (2004). Light-harvesting features revealed by the structure of plant Photosystem I. Photosyn. Res. 81, 239–250. 10.1023/B:PRES.0000036881.23512.42 16034530

[B9] Bokhari U. G. (1976). Influence of temperatures, water stress, and nitrogen treatments on chlorophyll and dry matter of western wheatgrass. J. Range Manage. 29, 127–131. 10.2307/3897408

[B10] Boussadia O. Steppe K. Zgallai H. Ben El Hadj S. Braham M. Lemeur R. (2011). Nondestructive determination of nitrogen and chlorophyll content in olive tree leaves and the relation with photosynthesis and fluorescence parameters. Photosynthetica 49, 149–153. 10.1007/s11099-011-0019-x

[B11] Brestic M. Zivcak M. Kunderlikova K. Sytar O. Shao H. Kalaji H. M. (2015). Low PSI content limits the photoprotection of PSI and PSII in early growth stages of chlorophyll *b*-deficient wheat mutant lines. Photosyn. Res. 125, 151–166. 10.1007/s11120-015-0093-1 25648638

[B12] Castrillo M. (2006). Fotosíntesis en tres poblaciones altitudinales de la planta andina *Espeletia schultzii* (Compositae). Rev. Biol. Trop. 54, 1143–1149. 10.15517/rbt.v54i4.3091 18457152

[B13] Croft H. Chen J. M. Froelich N. J. Chen B. Staebler R. M. (2015). Seasonal controls of canopy chlorophyll content on forest carbon uptake: implications for GPP modelling. J. Geophys. Res. Biogeosci. 120, 1576–1586. 10.1002/2015JG002980

[B14] Croft H. Chen J. M. Luo X. Bartlett P. Chen B. Staebler R. M. (2017). Leaf chlorophyll content as a proxy for leaf photosynthetic capacity. Global Change Biol. 23, 3513–3524. 10.1111/gcb.13599 27976452

[B15] Emilie E. Hutley L. B. Rossiter-Rachor N. A. Michael M. D. Samantha A. S. (2015). Resource-use efficiency explains grassy weed invasion in a low-resource savanna in north Australia. Front. Plant Sci. 6, 560. 10.3389/fpls.2015.00560 26300890 PMC4523779

[B16] Fajardo A. Piper F. II (2017). An assessment of carbon and nutrient limitations in the formation of the southern Andes tree line. J. Ecol. 105, 517–527. 10.1111/1365-2745.12697

[B17] Grieco M. Suorsa M. Jajoo A. Tikkanen M. Aro E. M. (2015). Lightharvesting II antenna trimers connect energetically the entire photosynthetic machinery - including both photosystems II and I. Biochim. Biophys. Acta 1847, 607–619. 10.1016/j.bbabio.2015.03.004 25843550

[B18] Gross N. Kunstler G. Liancourt P. Bello F. D. Suding K. N. Lavorel S. (2009). Linking individual response to biotic interactions with community structure: a trait-based framework. Funct. Ecol. 23, 1167–1178. 10.1111/j.1365-2435.2009.01591.x

[B19] Gross N. Bagousse-Pinguet Y. L. Liancourt P. Berdugo M. Gotelli N. J. Maestre F. T. (2017). Functional trait diversity maximizes ecosystem multifunctionality. Nat. Ecol. Evol. 1, 132. 10.1038/s41559-017-0132 28812705

[B20] Hallik L. Kull O. Niinemets Ü. Aan A. (2009). Contrasting correlation networks between leaf structure, nitrogen and chlorophyll in herbaceous and woody canopies. Basic Appl. Ecol. 10, 309–318. 10.1016/j.baae.2008.08.001

[B21] Hansen U. Fiedler B. Rank B. (2002). Variation of pigment composition and antioxidative systems along the canopy light gradient in a mixed beech/oak forest: a comparative study on deciduous tree species differing in shade tolerance. Trees: Struct. Funct. 16, 354–364. 10.1007/s00468-002-0163-9

[B22] Harpole W. Sullivan L. Lind E. Firn J. Adler P. Borer E. (2016). Addition of multiple limiting resources reduces grassland diversity. Nature 537, 93–96. 10.1038/nature19324 27556951

[B23] He J. S. Wang X. Schmid B. Flynn D. F. B. Li X. Reich P. B. (2010). Taxonomic identity, phylogeny, climate and soil fertility as drivers of leaf traits across Chinese grassland biomes. J. Plant Res. 123, 551–561. 10.1007/s10265-009-0294-9 20066555

[B24] Huang Z. A. Jiang D. A. Yang Y. Sun J. W. Jin S. H. (2004). Effects of nitrogen deficiency on gas exchange, chlorophyll fluorescence, and antioxidant enzymes in leaves of rice plants. Photosynthetica 42, 357–364. 10.1023/B:PHOT.0000046153.08935.4c

[B25] Huang M. Piao S. Janssens I. A. Zhu Z. Wang T. Wu D. (2017). Velocity of change in vegetation productivity over northern high latitudes. Nat. Ecol. Evol. 1, 1649–1654. 10.1038/s41559-017-0328-y 28970570

[B26] Ivanov A. G. Rosso D. Savitch L. V. Stachula P. Rosembert M. Oquist G. (2012). Implications of alternative electron sinks in increased resistance of PSII and PSI photochemistry to high light stress in cold-acclimated *Arabidopsis thaliana* . Photosyn. Res. 113, 191–206. 10.1007/s11120-012-9769-y 22843101

[B27] Jalota S. K. Vashisht B. B. Sharma S. Kaur S. (2018). “Chapter 3–Cimate change impact on crop productivity and field water balance,” in Understanding Climate Change Impacts on Crop Productivity and Water Balance (Salt lake city, UT, US: Academic Press), 87–148.

[B28] Kitajima K. Hogan K. P. (2003). Increases of chlorophyll a/b ratios during acclimation of tropical woody seedlings to nitrogen limitation and high light. Plant Cell Environ. 26, 857–865. 10.1046/j.1365-3040.2003.01017.x 12803613

[B29] Knapp A. K. Smith M. D. (2001). Variation among biomes in temporal dynamics of aboveground primary production. Science 291, 481–484. 10.1126/science.291.5503.481 11161201

[B30] Knapp A. K. Fay P. A. Blair J. M. Collins S. L. Smith M. D. Carlisle J. D. (2002). Rainfall variability, carbon cycling, and plant species diversity in a mesic grassland. Science 298, 2202–2205. 10.1126/science.1076347 12481139

[B31] Kull O. (2002). Acclimation of photosynthesis in canopies: models and limitations. Oecologia 133, 267–279. 10.1007/s00442-002-1042-1 28466225

[B32] Larkum A. W. D. Ritchie R. J. Raven J. A. (2018). Living off the Sun: chlorophylls, bacteriochlorophylls and rhodopsins. Photosynthetica 56, 11–43. 10.1007/s11099-018-0792-x

[B33] Leong T. Y. Goodchild D. J. Anderson J. M. (1985). Effect of light quality on the composition, function, and structure of photosynthetic thylakoid membranes of asplenium australasicum (Sm.) Hook. Plant Physiol. 78, 561–567. 10.1104/pp.78.3.561 16664283 PMC1064776

[B34] Li X. Xiao J. He B. Arain M. A. Beringer J. Desai A. R. (2018a). Solar-induced chlorophyll fluorescence is strongly correlated with terrestrial photosynthesis for a wide variety of biomes: First global analysis based on OCO-2 and flux tower observations. Global Change Biol. 24, 3990–4008. 10.1111/gcb.14297 29733483

[B35] Li Y. He N. Hou J. Xu L. Liu C. Zhang J. (2018b). Factors influencing leaf chlorophyll content in natural forests at the biome scale. Front. Ecol. Evol. 6, 64. 10.3389/fevo.2018.00064

[B36] Li Y. Liu C. Zhang J. Yang H. Xu L. Wang Q. (2018c). Variation in leaf chlorophyll concentration from tropical to cold-temperate forests: association with gross primary productivity. Ecol. Indic. 85, 383–389. 10.1016/j.ecolind.2017.10.025

[B37] Liu G. Freschet G. T. Pan X. Cornelissen J. H. C. Li Y. Dong M. (2010). Coordinated variation in leaf and root traits across multiple spatial scales in Chinese semi-arid and arid ecosystems. New Phytol. 188, 543–553. 10.1111/j.1469-8137.2010.03388.x 20649915

[B38] Liu C. Wang X. Wu X. Dai S. He J. S. Yin W. (2013). Relative effects of phylogeny, biological characters and environments on leaf traits in shrub biomes across central Inner Mongolia, China. J. Plant Ecol. 6, 220–231. 10.1093/jpe/rts028

[B39] Liu H. Hu B. Wang Y. Liu G. Tang L. Ji D. (2017). Two ultraviolet radiation datasets that cover China. Adv. Atmos. Sci. 34, 805–815. 10.1007/s00376-017-6293-1

[B40] Luo W. Elser J. J. Lv X. T. Wang Z. Bai E. Yan C. (2015). Plant nutrients do not covary with soil nutrients under changing climatic conditions. Global Biogeochem. Cycles 29, 1298–1308. 10.1002/2015gb005089

[B41] Lv X. Zhou G. Wang Y. Song X. (2016). Sensitive indicators of zonal *Stipa* Species to changing temperature and precipitation in Inner Mongolia grassland, China. Front. Plant Sci. 7, 73. 10.3389/fpls.2016.00073 26904048 PMC4744897

[B42] Murchie E. H. Horton P. (1997). Acclimation of photosynthesis to irradiance and spectral quality in British plant species: chlorophyll content, photosynthetic capacity and habitat preference. Plant Cell Environ. 20, 438–448. 10.1046/j.1365-3040.1997.d01-95.x

[B43] Pérez C. A. Armesto J. J. (2017). Coupling of microbial nitrogen transformations and climate in sclerophyll forest soils from the Mediterranean Region of central Chile. Sci. Total Environ. 625, 394–402. 10.1016/j.scitotenv.2017.12.306 29289787

[B44] Porra R. J. Scheer H. (2019). Towards a more accurate future for chlorophyll *a* and *b* determinations: the inaccuracies of Daniel Arnon’s assay. Photosyn. Res. 140, 215–219. 10.1007/s11120-018-0579-8 30194670

[B45] Reich P. B. Walters M. B. Ellsworth D. S. Uhl C. (1994). Photosynthesis-nitrogen relations in Amazonian tree species - I. Patterns among species and communities. Oecologia 97, 62–72. 10.1007/bf00317910 28313590

[B46] Schamp B. S. Chau J. Aarssen L. W. (2008). Dispersion of traits related to competitive ability in an old-field plant community. J. Ecol. 96, 204–212. 10.2307/20143455

[B47] Schwarz E. M. Tietz S. Froehlich J. E. (2018). Photosystem I-LHCII megacomplexes respond to high light and aging in plants. Photosyn. Res. 136, 107–124. 10.1007/s11120-017-0447-y 28975583 PMC5851685

[B48] Sekhar S. Panda D. Kumar J. Niharika M. Monalisha B. Mirza J. B. (2019). Comparative transcriptome profiling of low light tolerant and sensitive rice varieties induced by low light stress at active tillering stage. Sci. Rep. 9, 5753. 10.1038/s41598-019-42170-5 30962576 PMC6453891

[B49] Shao Q. Wang H. Guo H. Zhou A. Huang Y. Sun Y. (2014). Effects of shade treatments on photosynthetic characteristics, chloroplast ultrastructure, and physiology of *Anoectochilus roxburghii* . PloS One 9, e85996. 10.1371/journal.pone.0085996 24516523 PMC3917826

[B50] Shiflett S. A. Zinnert J. C. Young D. R. (2014). Coordination of leaf N, anatomy, photosynthetic capacity, and hydraulics enhances evergreen expansive potential. Trees 28, 1635–1644. 10.1007/s00468-014-1073-3

[B51] Sitaula B. K. Bakken L. R. (1993). Nitrous oxide release from spruce forest soil: relationships with nitrification, methane uptake, temperature, moisture and fertilization. Soil Biol. Biochem. 25, 1415–1421. 10.1016/0038-0717(93)90056-H

[B52] Stock W. D. Verboom G. A. (2012). Phylogenetic ecology of foliar N and P concentrations and N:P ratios across mediterranean-type ecosystems. Global Ecol. Biogeogr. 21, 1147–1156. 10.1111/j.1466-8238.2011.00752.x

[B53] Sun Y. Frankenberg C. Jung M. Joiner J. Guanter L. Köhler P. (2018). Overview of Solar-Induced chlorophyll Fluorescence (SIF) from the Orbiting Carbon Observatory-2: Retrieval, cross-mission comparison, and global monitoring for GPP. Remote Sens. Environ. 209, 808–823. 10.1016/j.rse.2018.02.016

[B54] Tan S. Wolfe G. R. Cunningham F. X. Gantt E. (1995). Decrease of polypeptides in the PS I antenna complex with increasing growth irradiance in the red alga *Porphyridium cruentum* . Photosyn. Res. 45, 1–10. 10.1007/BF00032230 24301374

[B55] Terashima I. Hikosaka K. (1995). Comparative ecophysiology of leaf and canopy photosynthesis. Plant Cell Environ. 18, 1111–1128. 10.1111/j.1365-3040.1995.tb00623.x

[B56] Valladares F. Niinemets Ü. (2008). Shade tolerance, a key plant feature of complex nature and consequences. Annu. Rev. Ecol. Evol. Syst. 39, 237–257. 10.1146/annurev.ecolsys.39.110707.173506

[B57] Vladimir I. M. Boris V. T. Michael A. B. Andrei A. M. Alexander N. T. (2016). Light acclimation of shade-tolerant and light-resistant *Tradescantia* species: induction of chlorophyll *a* fluorescence and P700 photooxidation, expression of PsbS and Lhcb1 proteins. Photosyn. Res. 130, 275–291. 10.1007/s11120-016-0252-z 27037825

[B58] Wang R. Yu G. He N. Wang Q. Zhao N. Xu Z. (2015). Latitudinal variation of leaf stomatal traits from species to community level in forests: linkage with ecosystem productivity. Sci. Rep. 5, 14454. 10.1038/srep14454 26403303 PMC4585881

[B59] Wright I. J. Reich P. B. Westoby M. Ackerly D. D. Baruch Z. Bongers F. J. J. M. (2004). The worldwide leaf economics spectrum. Nature 428, 821. 10.1038/nature02403 15103368

[B60] Wright I. J. Reich P. B. Cornelissen J. H. C. Falster D. (2005). Assessing the generality of global leaf trait relationships. New Phytol. 166, 485–496. 10.1111/j.1469-8137.2005.01349.x 15819912

[B61] Wyka T. Robakowski P. Ytkowiak R. (2008). Leaf age as a factor in anatomical and physiological acclimative responses of *Taxus baccata* L. needles to contrasting irradiance environments. Photosyn. Res. 95, 87–99. 10.1007/s11120-007-9238-1 17891474

[B62] Xie J. B. Xu G. Q. Jenerette G. D. Bai Y. F. Wang Z. Y. Li Y. (2015). Apparent plasticity in functional traits determining competitive ability and spatial distribution: a case from desert. Sci. Rep. 5, 12174. 10.1038/srep12174 26190745 PMC4507175

[B63] Yu Y. Jin Z. Lin H. Wang Y. Zheng H. (2019). Spatial variation and soil nitrogen potential hotspots in a mixed land cover catchment on the Chinese Loess Plateau. J. Mt. Sci. 16, 1353–1366. 10.1007/s11629-018-5175-z

[B64] Zhang C. Zhang D. W. Deng X. G. Tian Z. H. Lin H. H. (2019). Various adaptations of meadow forage grasses in response to temperature changes on the Qinghai–Tibet Plateau, China. Plant Growth Regul. 88, 181–193. 10.1007/s10725-019-00499-x

[B65] Zhao N. He N. Wang Q. Zhang X. Wang R. Xu Z. (2014). The altitudinal patterns of leaf C:N:P stoichiometry are regulated by plant growth form, climate and soil on Changbai Mountain, China. PloS One 9, e95196. 10.1371/journal.pone.0095196 24743878 PMC3990608

[B66] Zhao N. Yu G. He N. Xia F. Wang Q. Wang R. (2016a). Invariant allometric scaling of nitrogen and phosphorus in leaves, stems, and fine roots of woody plants along an altitudinal gradient. J. Plant Res. 129, 647–657. 10.1007/s10265-016-0805-4 26943163

[B67] Zhao N. Yu G. He N. Wang Q. Guo D. (2016b). Coordinated pattern of multi-element variability in leaves and roots across Chinese forest biomes. Global Ecol. Biogeogr. 25, 359–367. 10.1111/geb.1242

[B68] Zivcak M. Brestic M. Kalaji H. M. Govindjee (2014). Photosynthetic responses of sun- and shade-grown barley leaves to high light: is the lower PSII connectivity in shade leaves associated with protection against excess of light? Photosyn. Res. 119, 339–354. 10.1007/s11120-014-9969-8 24445618 PMC3923118

